# Decremental response in patients with amyotrophic lateral sclerosis during repetitive nerve stimulation and its relationships with impaired homeostasis

**DOI:** 10.3389/fnagi.2024.1502025

**Published:** 2025-01-07

**Authors:** Jinghong Zhang, Yang Li, Qiang Shi

**Affiliations:** Department of Neurology, The First Medical Center, Chinese PLA General Hospital, Beijing, China

**Keywords:** amyotrophic lateral sclerosis, repetitive nerve stimulation, decremental response, electromyography, modeling

## Abstract

**Background:**

Previous studies have suggested that neuromuscular junction (NMJ) denervation plays a critical role in amyotrophic lateral sclerosis (ALS). Repetitive nerve stimulation (RNS) has been used as a technique to test neuromuscular transmission, but the sensitivity and stability of its parameters have not been investigated in patients with ALS. In addition, the impact of impaired homeostasis on NMJ stability in patients with ALS remains unclear.

**Methods:**

A total of 421 patients with ALS were enrolled. Data on their clinical, biochemical and electrophysiological indicators were divided into a training set (collected from June 2019 to June 2022) and a test set (collected from July 2022 to June 2023). The coefficient of variation (CV) was used to assess the extent of variability. Stepwise regression was used in independent variable selection and model building.

**Results:**

In patients with ALS, area decrement had a higher rate of abnormal result and a lower CV than amplitude decrement. No significant difference in the rate of abnormal decrement was found when the first compound muscle action potential (CMAP) was compared with either the fourth or fifth one. Moreover, multivariate regression analysis suggests high-density lipoprotein cholesterol (HDL-C) had the greatest impact on decremental response, followed by serum uric acid (UA) and forced vital capacity (FVC). Females had a larger range of area decrement than males.

**Conclusion:**

During RNS test, assessing area decrement significantly enhances our ability to detect the impairment of neuromuscular transmission in patients with ALS. Independent factors contributing to decremental response need to be considered in drug development and clinical trials targeting NMJ in patients with ALS.

## Introduction

Amyotrophic lateral sclerosis (ALS) is the third most common adult-onset neurodegenerative disease. The incidence of ALS exponentially increases with age after 40, peaking in individuals aged 70–79 years (Ingre et al., 2015). The global number of ALS patients is projected to increase by 69% between 2015 to 2040 (Arthur et al., 2016).

Neuromuscular junction (NMJ) degeneration is an early event with complex and dynamic process of continuous denervation and new innervation in ALS (Martineau et al., 2018). Cellular and molecular alterations in presynaptic motor neuron, postsynaptic skeletal muscle, and terminal Schwann cells contribute to NMJ degeneration (Verma et al., 2022). In recent years, peripheral stabilization of NMJs and motor-units (MUs) has been proposed as a potential therapeutic target for patients with ALS (Alhindi et al., 2022; Verma et al., 2022; Martineau et al., 2018).

Repetitive nerve stimulation (RNS) is a widely utilized technique to test neuromuscular transmission. The sensitivity and stability of measurements during RNS test are important issues of concern in clinical practice. Over the past 30 years, most studies have evaluated the decremental response by measuring the reduction in peak-to-peak amplitude of the compound muscle action potential (CMAP) from the first to the fourth (Killian et al., 1994; Hatanaka et al., 2017; Fu et al., 2019) or fifth stimulus (Mori et al., 2016; Wang et al., 2017; Hu et al., 2018), while others have assessed it by analyzing changes in CMAP area (Wang et al., 2001; de Carvalho and Swash, 2019). However, the comparisons of these parameters have not been well investigated in patients with ALS.

Besides, accumulating evidence has suggested that homeostasis is impaired in patients with ALS, and several biochemical blood parameters that confirm the presence or progression of a change in body status, have been regarded as possible biomarkers for the prognosis of ALS (Kirk et al., 2019; Monov and Molodozhnikova, 2024; Aydemir et al., 2022; Cheng et al., 2021). However, the impacts of these changes on neuromuscular transmission have not been fully elucidated in patients with ALS.

Therefore, the aim of this study was to investigate the sensitivity and stability of parameters during the RNS test and to identify independent clinical and biochemical factors contributing to the NMJ stability.

## Materials and methods

### Participants

This study enrolled patients diagnosed as ALS based on the Awaji criteria (de Carvalho et al., 2008), and who had undergone LF-RNS tests on the accessory nerve at our hospital between June 2019 to June 2023. Exclusion criteria were as follows: (1) patients with a history of poliomyelitis, (2) patients with a spinal cord tumor, (3) patients with other diseases affecting the peripheral nerves, neuromuscular junction (NMJ), or muscles, and (4) patients with incomplete clinical information. This study was approved by the ethics committee of our hospital and written informed consent was obtained from each participant.

### Clinical data

Clinical information was collected at the time of diagnosis, including sex, age at onset, onset site, disease duration, diagnostic level, ALSFRS-R score (Cedarbaum et al., 1999), body mass index (BMI), forced vital capacity (FVC), and the results of RNS tests. The rate of disease progression (ΔFS) was calculated through the following formula: ΔFS = [48–ALSFRS-R score]/duration (month) (Labra et al., 2016).

Experienced neurologists followed up on the participants regularly every 6 months with a telephone call or a face-to-face interview until June 30, 2024. For a deceased patient, survival time was defined as the interval time between date of onset and date of death or tracheotomy, which was taken as the equivalent to death. For the surviving patients, survival time was calculated from date of onset to the date of the last follow-up visit (June 30, 2024). Patients who made no contact on two successive occasions were assigned to loss in the follow-up group.

### Laboratory tests

The electrodiagnostic studies were performed on a Keypoint workstation (31A06, Alpine Biomed ApS, Copenhagen, Denmark). Skin temperature over the examined muscle was maintained at 32°C or above throughout the entire period of measurement. Spontaneous activity was considered as present when reproducible trains were observed for at least 300 ms following needle insertion at >2 sites within the same muscle. Ten MUPs were measured in each muscle.

LF-RNS was performed on the side with the more severe symptom involvement, and its results were recorded from the trapezius muscle with stimulation of the accessory nerve, abductor digiti minimi (ADM) muscle for the ulnar nerve, and tibialis anterior muscle (TAM) for the peroneal nerve. For patients asymptomatic on both upper limbs, the RNS test was performed on the right side. A train of 10 3 Hz stimulus was delivered to the nerves, which was repeated three times at an interval of at least 30 s between each stimulus train. The peak-to-peak amplitude decrement and the difference in negative peak area from the first CMAP to the fourth/fifth one were measured and expressed as percentages. A decrement of 10% or more was considered to be abnormal (Kimura, 1989).

Venous blood was collected from the cubital vein of the patient early in the morning after an overnight fast. Biochemical indicators were measured in the department of biochemistry in our hospital. The serum interleukin-6 (IL-6) level was measured using a chemiluminescence immunoassay and with the Immulite 2000 chemiluminescence immunoassay analyzer (Siemens), according to the instructions of the manufacturer. The functional sensitivity (FS) of IL-6 was 2 pg./mL. The detection values of IL-6 lower than FS were recorded as <2 pg./mL. Referring to the methods reported in the previous literature, values lower than FS were calculated as 0.5 × FS without affecting the statistical results. Serum triglyceride (TG), total cholesterol (TC), high-density lipoprotein cholesterol (HDL-C), low-density lipoprotein cholesterol (LDL-C), serum uric acid (UA), serum creatinine, and total serum bilirubin (TSB) were measured using an autoanalyzer (Cobas 8,000 modular analyzer series; Roche Diagnostics, Basel, Switzerland).

The pulmonary function test (PFT) was performed by certified technicians in the pulmonary function laboratory of our hospital. Pulmonary ventilation was measured with a spirometer (MasterScreen Body; Jaeger, Wurzburg, Germany) and the measurement results were analyzed with computer software (Master Lab Manager V5.31.0 software; Jaeger). Each test was repeated three times, and the best result was chosen as the final result. All spirometric values were expressed as a percentage of the predicted values.

### Statistical analysis

MS-Excel 2016 (Microsoft, Redmond, Washington) and MATLAB R2019b (MathWorks, Natick, Massachusetts) were employed for statistical analysis. Measurements that were normally distributed were expressed as mean ± standard deviation (SD), and those non-normally distributed as quartile M (Q1, Q3) values. Medians were compared with the Mann–Whitney U test between two groups. Enumeration data were presented as the number of patients and percentages. The Chi-square test was used for comparison between different enumerative groups. Bonferroni correction was made to *p* values when several statistical tests were being performed simultaneously. The survival analysis was performed by Kaplan Meier method with a log-rank test. The significance level was set at *p* < 0.05.

The extent of variability both in amplitude decrement and area decrement between the first train and the second or third one was investigated by means of coefficient of variation (CV). As to the area decrement from the first CMAP to the fourth, the values of the differences between the first train and the second, as well as between the third one and the first, were used to derive the mean difference value. Standard deviation and CV were derived from the data set that included the mean difference values of all patients. The lower CV indicates greater stability.

Stepwise regression was used for model building. In model validation, the predicted number of patients with abnormal decrement was derived by calculation of the model based on data from patients with abnormal decrements. The predicted number of patients with normal decrements was derived by calculation based on data from patients with normal decrements. Consistency in the same results was calculated as (predicted number of patients with abnormal decrement + predicted number of patients with normal decrement)/(actual number of patients with abnormal decrement + actual number of patients with normal decrement) × 100%.

## Results

### Cohort characteristics

A total of 421 patients with ALS were enrolled in this study, all having undergone LF-RNS on the trapezius with stimulation on the accessory nerve. Details on the demographics of ALS patients are presented in Table 1. Of the 421 patients, 191 had also undergone LF-RNS on the ADM, and 54 on the TAM.

**Table 1:** Demographic information of ALS patients.

	Participants (*n* = 421)
Onset age, years (mean ± SD, range)	53.7 ± 10.7 (27–86)
Disease duration, months (median, IQR)	12 (8–22)
BMI, kg/m^2^ (median, IQR)	23.5 (21.5–25.6)
Sex	
Male *n* (%)	246 (58.4%)
Female *n* (%)	175 (41.6%)
Diagnosis	
Clinically definite ALS *n* (%)	303 (72.0%)
Clinically probable ALS *n* (%)	79 (18.8%)
Clinically possible ALS *n* (%)	39 (9.2%)
Disease onset	
Upper limb *n* (%)	192 (45.6%)
Lower limb *n* (%)	128 (30.4%)
Bulbar	101 (24.0%)
ALSFRS-R (median, IQR)	41 (36–43) (*n* = 187)
Disease progression rate (median, IQR)	0.54 (0.29–1.12) (*n* = 187)
HDL-C, mmol/L (median, IQR)	1.21 (1.01, 1.46) (*n* = 342)
LDL-C, mmol/L (median, IQR)	2.92 (2.25, 3.46) (*n* = 342)
TC, mmol/L (median, IQR)	4.55 (3.84, 5.20) (*n* = 342)
TG, mmol/L (median, IQR)	1.35 (1.02, 1.87) (*n* = 342)
IL-6, mmol/L (median, IQR)	1.00 (1.00, 3.23) (*n* = 271)
TSB, pg./ml (median, IQR)	41.2 (39.5, 43.5) (*n* = 354)
Serum creatinine, μmol/L (median, IQR)	5.25 (4.32, 6.32) (*n* = 310)
Serum UA, μmol/L (median, IQR)	60.2 (52.2, 69.2) (*n* = 358)

**Figure 1:**
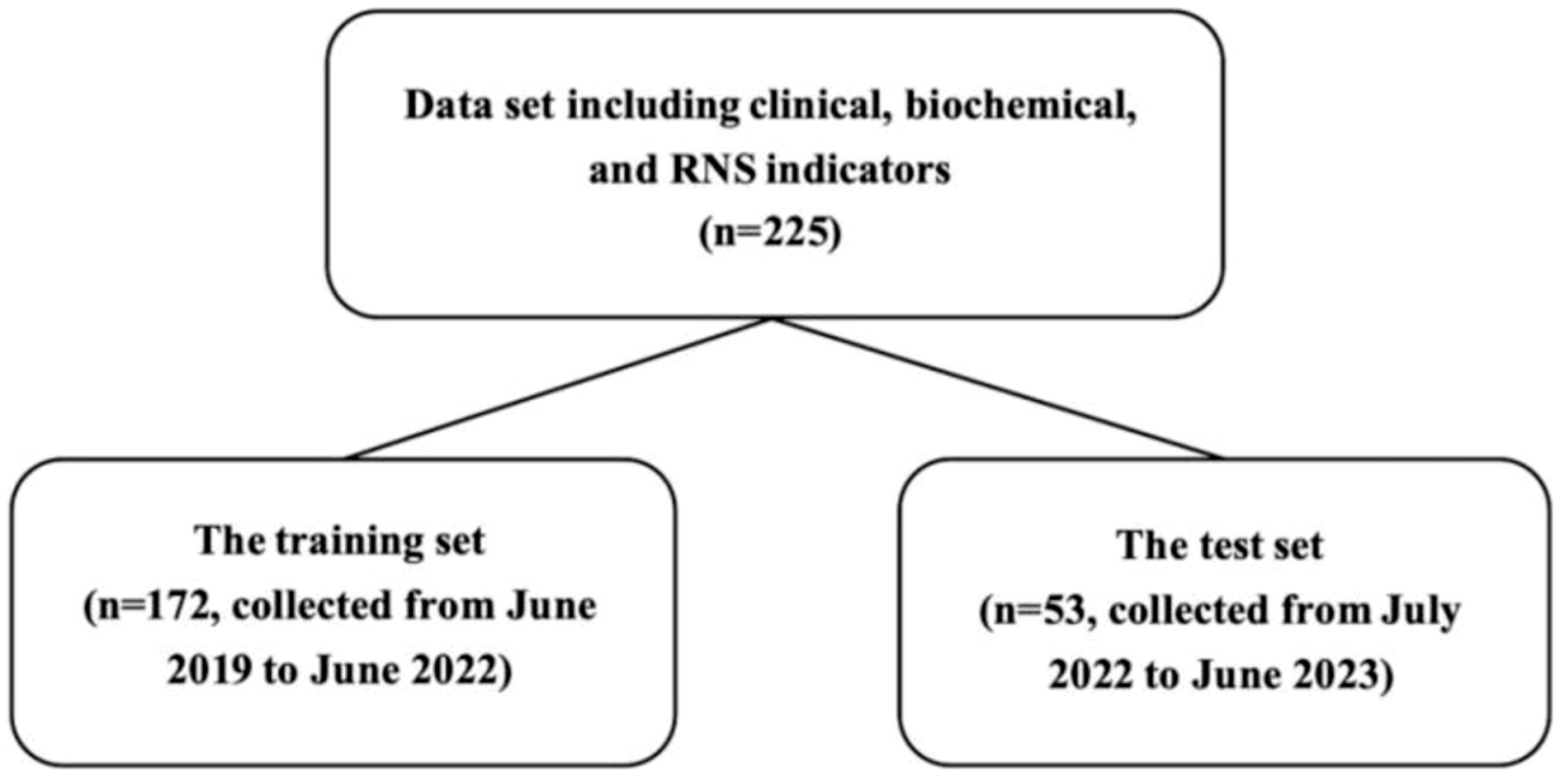
Data splitting. The flow diagram indicates the numbers in the training set and the test set.

Two hundred and eight patients attended follow-up visits between June 2019 and June 2024. One hundred and five patients had died or undergone tracheostomy, and 103 were still alive at the last follow-up visit. A total of 225 patients have complete records of biochemical tests. For the multivariate model building and validation, their clinical data were divided into a training set and a test set (Figure 1).

### Rate of abnormal amplitude decrement and area decrement during LF-RNS

The rate of abnormal decrement in trapezius is higher than that in ADM and TAM (Table 2). No significant difference was found in the abnormal rate of either amplitude or area decrement when the first CMAP is compared with either the fourth or fifth one.

**Table 2:** Amplitude decrement and area decrement during RNS at 3 Hz.

Muscles	Onset site	Percentage of cases with decrement ≥10%					
		Amp 1–4	Amp 1–5	*χ*^2^/P	Area1-4	Area1-5	*χ*^2^/P
Trapezius	Upper limb onset (*n* = 192)	52.6	57.5	0.752/0.386	76.0	80.6	0.952/0.329
	Other onset sites (*n* = 229)	31.4	32.8	0.085/0.771	61.6	62.8	0.070/0.792
ADM	Upper limb onset (*n* = 89)	9.0	11.5	0.296/0.587	36.0	25.6	2.062/0.151
	Other onset sites (*n* = 110)	2.8	3.3	0.051/0.821	18.8	17.9	0.033/0.856
TAM	Lower limb onset (*n* = 25)	0.0	6.7	–	43.8	26.7	1.998/0.157
	Other onset sites (*n* = 29)	0.0	0.0	–	26.3	21.1	0.017/0.897

ADM, abductor digiti minimi; TAM, tibialis anterior muscle; Amp 1–4, the decrement that is worked out by calculation of the amplitude difference between first CMAP and the fourth CMAP.

The abnormal rate in area decrement is higher than that in amplitude decrement, and 98.8% of the patients with an abnormal amplitude decrement in the accessory nerve also had abnormal decrement measured by the area. However, of the 287 patients with an abnormal area decrement between the first CMAP and the fourth at 3 Hz on the trapezius, 169 (58.9%) had an abnormal amplitude decrement. Of the 248 patients with a normal amplitude decrement, 118 (47.6%) had abnormal area decrement.

### The stability of amplitude decrement and area decrement

Overall, the CV of the area decrement is lower than that of the amplitude decrement (Table 3). Specifically, as far as the decrement response from the first CMAP to fourth CMAP on the trapezius during 3 Hz RNS is concerned, the mean value of CV for area decrement (0.995) is lower than that for amplitude decrement (1.073). The mean absolute differences between stimulus trains in amplitude decrement is significantly different from that in area decrement (Supplementary Table 4). Similar results can be found on ADM and TAM. As such, CMAP area decrement is more stable than amplitude decrement.

**Table 3:** Coefficient of variation for the decrement in 3 Hz RNS tests.

Muscle	*n*	Amp 1–4		Amp 1–5		Area 1–4		Area 1–5	
		x̄ ± s	CV	x̄ ± s	CV	x̄ ± s	CV	x̄ ± s	CV
Trapezius	421	2.345 ± 2.516	1.073	2.369 ± 2.486	1.050	3.594 ± 3.578	0.995	3.863 ± 3.908	1.012
ADM	199	1.829 ± 2.176	1.189	2.343 ± 3.719	1.587	4.730 ± 5.187	1.097	4.866 ± 6.370	1.309
TAM	54	3.932 ± 3.344	0.851	3.608 ± 4.846	1.343	5.122 ± 4.027	0.786	7.945 ± 8.578	1.080

x̄ ± s, mean ± standard deviation; CV, coefficient of variation (%); ADM, abductor digiti minimi; TAM, tibialis anterior muscle; Amp 1–4, the decrement that is worked out by calculation of the amplitude difference between first CMAP and the fourth CMAP.

### The relationship between the decrement and the results of needle EMG tests in ALS

A total of 345 patients underwent 3 Hz RNS of the accessory nerve and needle EMG of the ipsilateral trapezius. A total of 48 patients underwent 3 Hz RNS of the peroneal nerve and needle EMG of the ipsilateral TAM. CMAP decrement is correlated with MUP duration and amplitude (Table 4). Patients with fibrillation potentials and/or positive sharp waves (fib-psw) in trapezius have a larger decrement range than those without fib-psw (Table 5).

**Table 4:** Correlations between area decrement and MUP.

Muscle	CMAP decrement (%)	MUP duration (ms)	*r*/*P*	MUP amplitude (mV)	*r*/*P*
Trapezius(*n* = 345)	−8.6(−15.4, −4.6)	13.5 (11.7, 14.8)	−0.442/<0.001	1072 (614, 1485)	−0.346/<0.001
TAM (*n* = 48)	0.0 (−2.6,2.0)	16.0 (15.4, 16.5)	−0.276/0.046	1857 (1300, 2888)	−0.316/0.039

TAM, Tibialis anterior muscle.

**Table 5:** Correlations between area decrement and abnormal muscle fiber potentials.

	CMAP decrement in patients with fib-psw	CMAP decrement in patients without fib-psw	*z*/*P*
Trapezius (*n* = 345)	−14.3 (−19.5, −7.1)	−7.1 (−12.1, −3.5)	4.513/<0.001
TAM (*n* = 48)	−0.7 (−2.7, 2.0)	0.4 (−2.0, 2.7)	−4.254/0.049

TAM, Tibialis anterior muscle; fib-psw, fibrillation potentials and/or positive sharp waves.

### Associations between area decrement and multiple parameters in ALS

Since no significant difference was found in the abnormal rate of amplitude or area decrement when the first CMAP is compared with either the fourth or fifth one and the number of patients having abnormal decrement on ADM and TAM was limited, we took the decrement from the first stimuli to the fourth on the trapezius as an example to further investigate the correlations between clinical indicators and the rates of abnormal decrement.

Seven clinical indicators (onset age, sex, onset site, diagnostic level, diagnostic delay, BMI, FVC), and nine biochemical indicators (HDL-C, LDL-C, LDL/HDL, IL-6, TC, TG, TSB, serum creatinine, and serum UA) were considered as components that might contribute to model building. The final results of the stepwise regression (Supplementary Table 1) showed that the following model provided the best fit for the data set:


(1) $$\begin{array}{l} \exp.{\left(Ys\right)}^{0.30}=-0.5972+0.1217\ln\left(X_{1}\right)\\ +0.0837D_{1}+0.0973D_{2}+0.1790D_{3}\\ -0.1547\ln\left(X_{2}\right)+0.2487\ln\left(X_{3}\right) \end{array}$$

Where *Ys* is the standardization of area decrement (*Y*), *X_1_* is FVC, *X*_2_ is HDL-C, *X*_3_ is serum UA, D_1_ = 0 if female, D_1_ = 1 if male. (D_2_, D_3_) represents onset site: upper limb onset = (0, 0), lower limb onset = (1, 0), and bulbar onset = (0, 1). The *F*-value is 5.713 (*p* < 0.001, *n* = 172), Durbin Watson test (DW test) statistic value is 2.029 and the corresponding *p*-value is 0.5729. *T*-values for each parameter reach significant level (*p* < 0.05).

The total consistency between the actual value and the prediction value is 75.581%, where the discrepancy mainly occurs when the decrement is <10% (Table 6). Leave-one-out cross-validation and external validation (Supplementary Table 2) suggest a good and stable prediction performance of model (1).

**Table 6:** Consistency between prediction value and actual value in the model.

	Number of patients	Absolute values of residuals (x̄ ± s)	Consistency between actual results and predictions (%)
Decrement ≥15%	79	8.329 ± 10.286	91.139 (72/79)
10% ≤ decrement <10%	46	3.129 ± 2.135	91.304 (42/46)
Decrement <10%	47	7.145 ± 3.728	34.043 (16/47)
Total	172	6.615 ± 3.609	75.581 (130/172)

**Figure 2:**
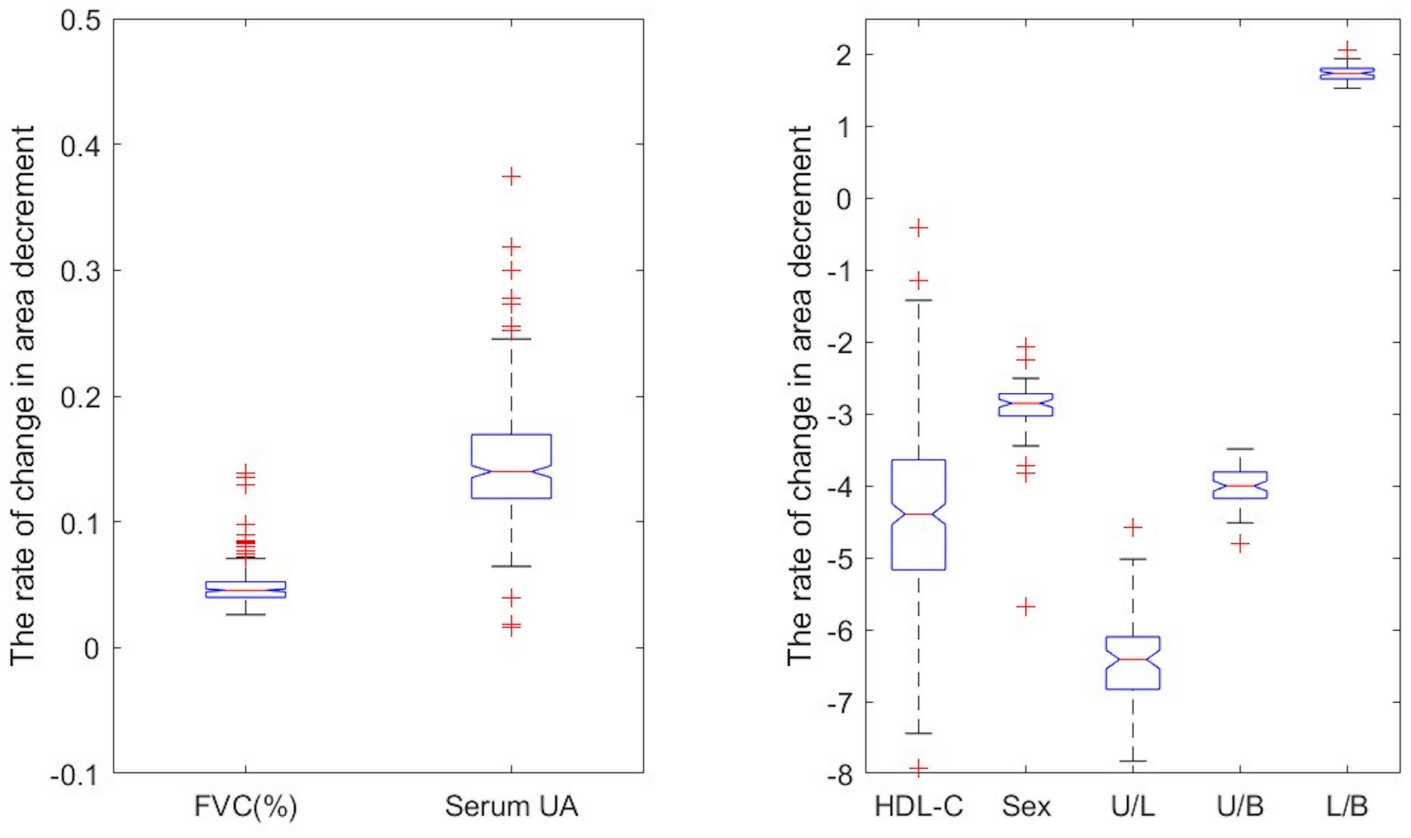
Predictor impact on the rate of decrement change. The U/L represents the difference in the rate of decrement change between upper limb onset and lower limb onset when holding other predictors constant.

FVC, HDL-C, serum UA, sex, and onset site are independent factors contributing to the area decrement (Figure 2). The model (1) suggests that with all other variables being held constant, a one unit increase in FVC (% predicted), HDL-C, or serum UA, were, respectively, associated with a 0.066, −4.4351, or 0.147% decreases in the range of area decrement (Supplementary Table 3). The expected range of area decrement in males was 2.9% lower than that in females. For patients with lower limb onset and bulbar onset, the expected ranges of area decrement decreased by 6.565 and 3.988%, respectively, in comparison with patients with upper limb onset. Therefore, HDL-C had the greatest impact on decremental response as measured by CMAP area, followed by serum UA and FVC; females had a larger range of area decrement than males; upper limb onset contributed more to the decremental response in accessory nerve than lower limb onset and bulbar onset.

### Clinical characteristics of patients having abnormal decrement only in CMAP area

Notably, in our cohort, there are 118 patients having abnormal decrement in CMAP area but normal decrement in amplitude. To find out their clinical characteristics, we compared them with patients having abnormal decrement in both measurements (Table 7). The results suggested that the patients with abnormal decrement only in area have shorter disease duration, higher ALSFRS-R scores, and a larger percentage of non-definite ALS and upper limb onset. No statistical difference in sex, onset age, BMI, FVC (%), and disease progression rate was observed.

**Table 7:** Comparison of clinical indicators between patients with normal amplitude decrement but abnormal area decrement and those having abnormal decrement in both measurements.

Clinical indicators		Total case number	Patients with amplitude decrement (−) and area decrement (+)		Patients with amplitude decrement (+) and area decrement (+)		*P*
			*n*	Median value	*n*	Median value	
Gender	Male	163	64	–	99	–	0.465
	Female	124	54	–	70	–	
Onset site	Upper limb onset	146	48	–	98	–	0.015
	Lower limb onset	80	39	–	41	–	
	Bulbar onset	61	31	–	30	–	
Diagnostic level	Definite ALS	222	83	–	139	–	0.018
	Non-definite ALS	65	35	–	30	–	
Onset age (year)		287	118	56.0 (48.0, 63.0)	169	56.0 (46.0, 63.0)	0.689
Disease duration (month)		287	118	11 (7, 20)	169	12 (9, 23)	0.042
BMI		287	118	23.7 (21.8, 25.7)	169	23.3 (21.5, 25.5)	0.846
FVC (%)		230	93	88.6 (75.5, 98.8)	137	89.7 (77.2, 97.5)	0.703
ALSFRS-R		135	51	41 (37, 44)	84	39 (34, 42)	0.048
∆FS (point/month)		135	51	0.50 (0.33, 0.98)	84	0.62 (0.31, 1.33)	0.521

### The impact of decremental response during LF-RNS on survival

**Figure 3:**
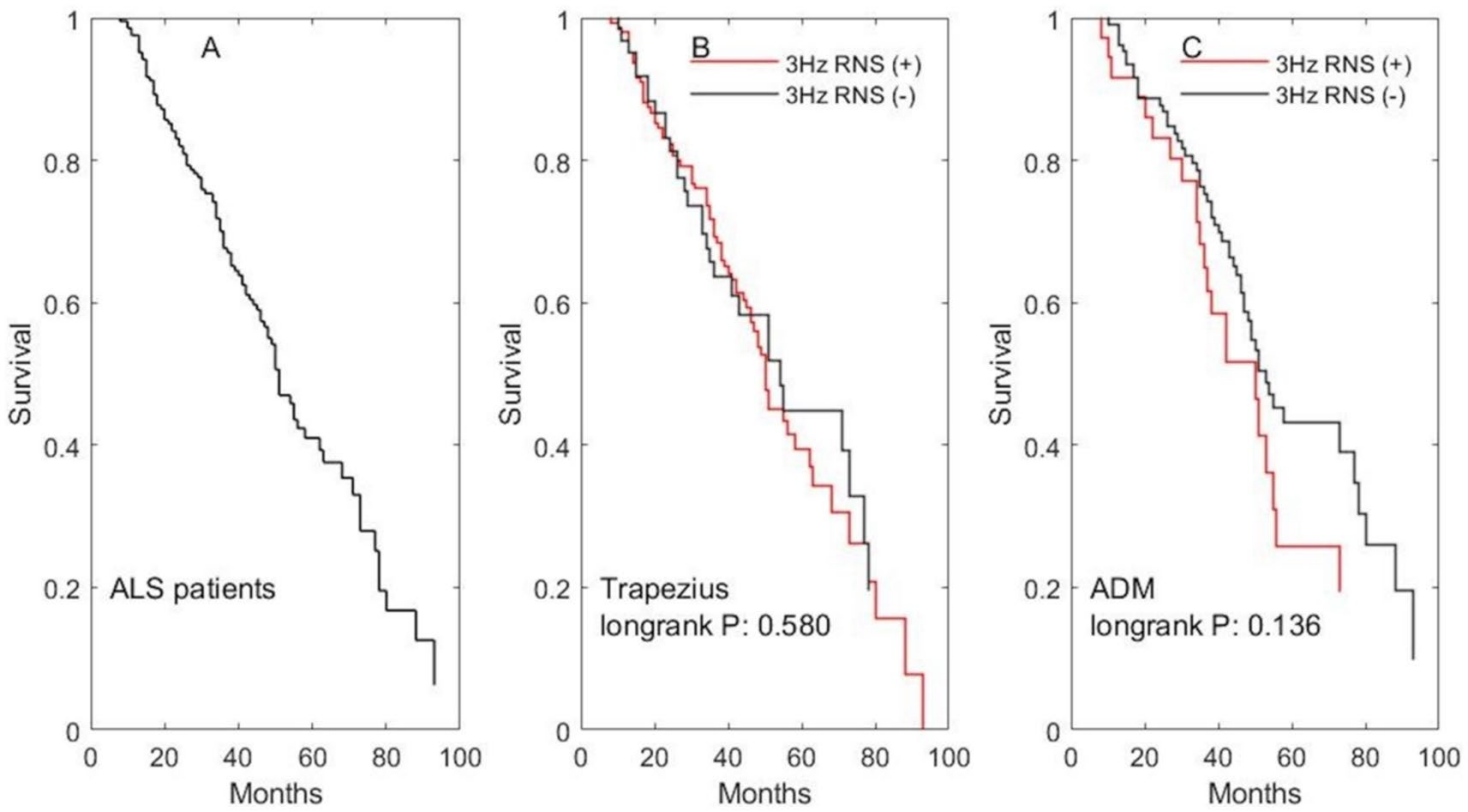
The impact of abnormal decremental response during 3 Hz RNS on survival. **(A)** The survival curve for all ALS patients. **(B)** The survival curves for patients undergone 3 Hz RNS test on the accessory nerve. **(C)** The survival curves for patients undergone 3 Hz RNS test on the ulnar nerve.

Finally, we analyzed the correlation between decremental response and survival. The mean survival time was 56.2 months (median: 53; 95%CI: 51.6–60.8) in our cohort. The median and mean survival time in patients with abnormal area decrement during 3 Hz RNS on the trapezius or ADM is shorter than that in patients with normal area decrement, but it did not reach statistical significance (Figure 3).

## Discussion

Our study showed that in patients with ALS, area decrement had a higher rate of abnormal result and a lower CV than amplitude decrement. No significant difference in the rate of abnormal decrement was found when the first CMAP was compared with either the fourth or fifth one. HDL-C, FVC, serum UA, sex, and onset site were independent factors contributing to the area decrement. Patients with abnormal decrement only in area had shorter disease duration, higher ALSFRS-R scores, and higher percentage of non-definite ALS than those having abnormal decrement in both measurements.

So far, the precise mechanism leading to CMAP decrement in ALS remains unclear. Previous studies have suggested that deregulation of protein homeostasis, malfunctioning of axonal transportation, cholinergic dysfunction, and impaired mitochondrial dynamics resulting from motor neuron degeneration contribute to NMJ disruption in ALS (Verma et al., 2022). On the other hand, inherent pathological defects in skeletal muscle independently induce NMJ degeneration (Badu-Mensah et al., 2022). ALS muscle cells fail to form large clusters of acetylcholine receptors (Shefner et al., 2023), and they secret excessive Nogo-A (Jokic et al., 2005; Bruneteau et al., 2015). In addition, perisynaptic Schwann cells fail to adopt a phagocytic phenotype on denervated NMJ and to guide compensatory nerve terminal sprouts (Martineau et al., 2020). In this study, correlations of decremental response with abnormal spontaneous activity and MUP have been observed, suggesting that both denervation and re-innervation influence CMAP decrement in patients with ALS.

It is generally assumed that the greatest decrease of acetylcholine release at a slow rate of stimulation occurs during the first four stimuli, and by the fifth or sixth stimulation, the release begins to improve because the mobilization store has begun resupplying the immediately available store (Preston and Shapiro, 2020). The percentage of the decrement is measured between the baseline CMAP and lowest CMAP. Fu et al. (2019) observed the descent pattern of CMAP amplitude in patients with ALS and found that during 3 Hz RNS, the lowest CMAP in the accessory nerve occurred at the 4^th^ stimulation, and that in ulnar nerve was at the 5th. Some researchers have calculated the decrement by comparing baseline CMAP with the CMAP amplitude from the fourth stimulus (Killian et al., 1994; Hatanaka et al., 2017; Fu et al., 2019), while others have chosen the fifth’s (Mori et al., 2016; Wang et al., 2017; Hu et al., 2018). Our study suggests that there is no statistical difference between the two calculation approaches, not only in the aspect of CMAP amplitude but also area.

CMAP area, the product of amplitude and duration, is known to be related most directly to the number of muscle fibers or motor units that contribute to the CMAP (Daube and Rubin, 2009; Asawa et al., 2004). In healthy volunteers, amplitude increases while duration decreases with successive low-frequency stimulation (Asawa et al., 2004; van Dijk et al., 2000). The impact of low-frequency stimulus on CMAP amplitude and duration may counteract each other in calculating CMAP area. In patients with ALS, cellular and molecular alterations in motor neurons, skeletal muscles, and terminal Schwann cells affect neuromuscular transmission (Verma et al., 2022), leading to fewer individual muscle fiber action potentials (MFAPs) contributing to the CMAP through four or five repetitions of low-frequency stimulus. Our study shows that, even when decremental response occurs, parameters of area decrement are more stable than amplitude decrement.

On the other hand, area decrement is more sensitive than amplitude decrement. In our cohort, 98.8% of the patients with abnormal amplitude decrement in the accessory nerve also had abnormal decrement measured by area, while 47.6% of patients with normal amplitude decrement had abnormal area decrement. Similar results were also found in patients with other neuromuscular disorders. Lo et al. (2003) compared area and amplitude of muscle responses to RNS in 87 patients with possible neuromuscular transmission defects and 30 controls. They found that decrement of response area provides additional diagnostic yields of up to 30%. Boonhong (2009) suggested that 23% of RNS reports were negative for amplitude decrement while positive for area decrement. Sixty-nine percent of those patients with inconsistent results were diagnosed with MG or suspected MG by the specialists. Therefore, area decrement greatly improves our ability to evaluate neuromuscular transmission. Moreover, patients with abnormal decrement only in area have a shorter disease duration, higher ALSFRS-R scores, and a larger percentage of non-definite ALS, thus indicating an earlier stage of ALS. This finding supports the suggestion by Ratnagopal (Ratnagopal, 1997) that area decrement may be a helpful indicator of neuromuscular defect at an earlier level.

In addition to the evaluation of the parameters during RNS test, we also investigated the relationship between decrement response and biochemical indicators in the present study. We found that decrement decreases with the increase of the natural antioxidant (serum uric acid), which is compatible with previous studies suggested that NMJ is very sensitive reacting to oxidative stress in ALS (Naumenko et al., 2011; Pollari et al., 2014). Besides, cholesterol is included in all key pre- and postsynaptic processes and plays a crucial role in safety factor at the NMJ (Krivoi and Petrov, 2019). Higher pre-diagnostic HDL-C levels have been found to be associated with a higher risk of ALS (Bjornevik et al., 2021). Statin, a widely used medication that reduces the level of LDL and raises the concentration of HDL (Al-Kuraishy et al., 2024; Barter et al., 2010), is associated with higher incidence (Wang et al., 2023; Skajaa et al., 2021), worse progression and shorter survival of ALS (Zinman et al., 2008; Weisskopf et al., 2022). Thus far, much less is known about how the lipid profile impacts the neuromuscular transmission in patients with ALS. The regression analysis in our study suggests that total cholesterol, LDL-C, and the LDL-C/HDL-C ratio are not independent factors contributing to the decremental response, while the range of decrement increases with HDL-C. The causal relationship between HDL-C elevation and NMJ dysfunction and whether it is an appropriate therapeutic target to improve neuromuscular transmission and produce effective relief for muscle weakness, still need further investigation.

There are some limitations to this study. First, we did not analyze the correlation of survival with decremental response on TAM because of the low abnormal rate and limited number of patients. Second, the number of patients who undergwent EMG test on the ADM is limited in our cohort. The relationship between the decrement and the results of needle EMG tests on the ADM needs further investigation. Third, in addition to the nine biochemical parameters discussed in current study, the relationships between decremental response and other aberrant biochemical parameters need further investigation. Forth, our patients are from different parts of China and are representative only of the ALS cohort in China. The findings of this study cannot be generalized and multicenter retrospective studies are needed in the future to determine whether there are any differences in the stability and sensitivity of measurements during LF-RNS between Chinese cohorts and patients from other countries or regions.

In conclusion, CMAP area decrement is more stable and sensitive than amplitude decrement in patients with ALS. Independent clinical and biochemical factors contributing to the decremental response should be considered in drug development and clinical trials targeting the NMJ in these patients.

## Data Availability

The data analyzed in this study is subject to the following licenses/restrictions: The data that support the findings of this study are available from the corresponding author upon reasonable request. Requests to access these datasets should be directed to Qiang Shi, shiq301@163.com.
