# Supplementary material for: Decremental response in patients with amyotrophic lateral sclerosis during repetitive nerve stimulation and its relationships with impaired homeostasis
Source: Front Aging Neurosci. 2025 Jan 7;16:1502025. doi: 10.3389/fnagi.2024.1502025 (PMC11747797; doi:10.3389/fnagi.2024.1502025)
Supplement: Supplementary file 1 [file Table_1.docx]

**Supplementary Material**

**Decremental response in patients with amyotrophic lateral sclerosis during repetitive nerve stimulation and its relationships with impaired homeostasis**

Jinghong Zhang ^1^; Yang Li ^1^; Qiang Shi ^1*^

^1^ Department of Neurology, the First Medical Center, Chinese PLA General Hospital, Beijing, 100853, China

1. **Formula development of area decrement (%) in the RNS test on the accessory nerve (Y)**
2. **Assessment of relationship strength between the quantitative variables and the area decrement at 3Hz RNS test on the accessory nerve**
3. **Decrement differences in qualitative variables**
4. **STable 1: The results of stepwise regression.**
5. **STable 2: Internal and external validation of prediction model.**
6. **STable 3: Predictor impact on the rate of decrement change.**
7. **STable 4: The mean absolute differences of the decrement in RNS tests.**
8. **Formula development of area decrement (%) in the RNS test on the accessory nerve (Y)**

In the current study, *Y* represents the area decrement (%) in the 3Hz RNS test of the accessory nerve. Because it significantly deviated from a normal distribution, we standardized *Y* as *Y*s by Z-score standardization, making it easier to use for model building:

(1)

where $\bar{y}$ represents the mean value of the decrement percentage in the dataset of the RNS test results and S_y_ represents the standard deviation. In the dataset of this study, the mean value of area decrement is -15.6% and the standard deviation is 10.7%.

After numerous computations and comparisons over many times, the natural logarithm of both *Ys* and predictor values provided the best fit for the prediction model:

$\text{exp(}Y\text{s)}^{\text{h}}\text{=b}_{0}+\text{b}_{1}\text{ln}\left(X_{1}\right)+\text{b}_{2}D_{1}+\text{b}_{3}D_{2}+\text{b}_{4}D_{3}+\text{b}_{5}\text{ln}\left(X_{2}\right)+\text{b}_{6}\text{ln}\left(X_{3}\right)+\delta.$ (2)

where *Y*s represents the standardization score of the area decrement in the 3Hz RNS test on the accessory nerve, *X*_1_, *X*_2_, and *X*_3_ represents the FVC (%), HDL-C (mmol/L), and serum UA (μmol/L), respectively. D_1_ represents sex and is coded 0 for men and 1 for women. D_2_ and D_3_ are dummy variables representing the onset site. Upper limb onset was coded (0,0), lower limb onset as (1, 0), and bulbar onset as (0, 1). h, b_0_-b_6_ are model parameters, and δ is the errors of the estimate caused by factors that have not been considered in the model.

*Y*m represents the predicted outcome of formula (2)

Therefore,

*Y*m=exp(*Y*s) ^h^

$$Y\text{s}=\frac{1}{\text{h}}\ln(Y\text{m})$$

Finally,

(3)

1. **Assessment of relationship strength between the quantitative variables and the area decrement at 3Hz RNS test on the accessory nerve**

When holding other predictor variables constant in the equation (2)

and since *Y*m = exp(*Y*s) ^h^, we derive

$$\frac{\text{d}Y\text{m}}{\text{d}X}=\text{b}\frac{1}{X}$$

where *Y*m represents the predicted outcome of area decrement, *X* represents the value of the patient’s quantitative indicator and b is the corresponding coefficient.

$$\text{d}Y\text{m }=\text{ dexp}\left(Y\text{s}\right)^{\text{h}}=\text{h exp}\left(Y\text{s}\right)^{\text{h}-1}\text{dexp}\left(Y\text{s}\right)=\text{h exp}\left(Y\text{s}\right)^{\text{h}-1}\text{exp}\left(Y\text{s}\right)\text{d}Y\text{s}=\text{h exp}\left(Y\text{s}\right)^{\text{h}}dY\text{s}.$$

Rearranging the equation (1), we get

Therefore,

Combining equations mentioned above, the following is obtained:

Since$\text{exp}\left(Y\text{s}\right)^{\text{h}}=Ym$, we get

1. **Decrement differences in qualitative variables**
2. Differences in sex:

Rearranging equation (1), we get:.

Since $\exp(Y\text{s})^{\mathrm{h}}=Y\text{m, h}Y\text{s = ln(}Y\text{m)}$, we get

$$\text{ }Y\text{s = }\frac{1}{\text{h}}\ln(Y\text{m})$$

$Ym_{man}-Ym_{woman}=\text{exp}(Ys_{\text{man}})^{h}-\text{ }exp(Ys_{\text{woman}})^{h}=-b_{2.}$*,Y*m_man_ = -*b*_2_ + *Y*m_woman_

Therefore,

Finally,

(4)

1. Differences in onset sites:

Since

we get

Since

,

we get

(5)

Similarly, the difference between the upper limb onset and bulbar onset is

(6)

The difference between the lower limb onset and bulbar onset is

(7)

Overall, the equations (4), (5), (6) and (7) can be summarized as:

where b=b_2_ and *Y*m represents the prediction outcome of female decrement when comparing the decrement between male and female patients; b=b_3_ and *Y*m represents the prediction outcome of lower limb onset when comparing the decrement between upper limb onset and lower limb onset; b=b_4_ and *Y*m represents the prediction outcome of bulbar onset when comparing the decrement between upper limb onset and bulbar onset; b=b_3_-b_4_ and *Y*m represents the prediction outcome of bulbar onset when comparing the decrement between lower limb onset and bulbar onset.

**4. STable 1: The results of stepwise regression**

STable 1. The results of stepwise regression.

| Indicators | Coefficient | SD | Status | *P* |
| --- | --- | --- | --- | --- |
| Age at onset | 0.0804 | 0.0816 | Exclude | 0.3266 |
| Sex (D_1_) | 0.0837 | 0.0413 | Include | 0.0445 |
| Onset site (D_2_) | 0.0973 | 0.0403 | Include | 0.0169 |
| Onset site (D_3_) | 0.1790 | 0.0473 | Include | 0.0002 |
| Diagnostic level (D_4_) | 0.0270 | 0.0398 | Exclude | 0.4975 |
| Disease duration | -0.0161 | 0.0246 | Exclude | 0.5126 |
| BMI | -0.0598 | 0.1364 | Exclude | 0.6617 |
| FVC | 0.1217 | 0.0489 | Include | 0.0138 |
| HDL-C | -0.1547 | 0.0530 | Include | 0.0040 |
| LDL-C | 0.0477 | 0.0569 | Exclude | 0.4023 |
| LDL-C/HDL-C | 0.0640 | 0.0534 | Exclude | 0.2323 |
| IL-6 | -0.0013 | 0.0207 | Exclude | 0.9519 |
| TC | 0.0538 | 0.0462 | Exclude | 0.2459 |
| TG | 0.0154 | 0.0442 | Exclude | 0.7289 |
| TSB | -0.0045 | 0.2496 | Exclude | 0.9857 |
| Serum creatinine | -0.0115 | 0.0687 | Exclude | 0.8677 |
| Serum UA | 0.2487 | 0.07912 | Include | 0.0020 |
| Intercept | -0.5972 | - | - | - |

Note: D_1_ represents sex and is coded 0 for men and 1 for women. D_2_ and D_3_ are dummy variables representing the onset site. Upper limb onset was coded (0,0), lower limb onset as (1, 0), and bulbar onset as (0, 1). SCM: sternocleidomastoid. TRA represents trapezius muscle. SD = standard deviation.

**5. STable 2: Internal and external validation of prediction model.**

STable 2. Internal and external validation of prediction model.

| Validation approach | Numbers of patients | Absolute values of residuals ($\bar{x}$± s) | Actural number of patients with abnormal decrement | Predicted number of patients with abnormal decrement | Actual number of patients with normal decrement | Predicted number of patients with normal decrement | Consistency in the same results (%) |
| --- | --- | --- | --- | --- | --- | --- | --- |
| Leave-one-out cross-validation | 172 | 7.227±3.423 | 125 | 114 | 47 | 12 | 73.256 |
| External validation | 53 | 7.457±10.846 | 37 | 33 | 16 | 5 | 71.698 |

**6. STable 3: Predictor impact on the rate of decrement change.**

STable 3. Predictor impact on the rate of decrement change.

| **Predictor** | | $\bar{x}\pm s$ | **Quartile** |
| --- | --- | --- | --- |
| FVC (%) | | 0.066±0.293 | 0.046 (0.040, 0.052) |
| HDL-C (mmol/L) | | -4.435±1.249 | -4.394 (-5.169, -3.638) |
| Serum UA (μmol/L) | | 0.147±0.047 | 0.140 (0.119, 0.169) |
| Sex | Male-female | -2.913±0.419 | -2.853 (-3.029, -2.720) |
| Onset site | Upper limb-lower limb | -6.565±1.028 | -6.832 (-6.832, -6.101) |
|  | Upper limb-bulbar | -3.988±2.570 | -4.002 (-4.176, -3.807) |
|  | Lower limb-bulbar limb | 1.731±0.103 | 1.737 (1.659, 1.806) |

Note: The mean and median values of the rate of decrement change were similar, indicating a normal distribution. Male-female represents the difference between male and female in the rate of decrement change when holding other predictors constant. The upper limb-lower limb represents the difference in the rate of decrement change between upper limb onset and lower limb onset when holding other predictors constant.

**7. STable 4: The mean absolute differences of the decrement in RNS tests.**

STable 4: The mean absolute differences of the decrement in RNS tests.

| Muscle | *n* | The decrement from the first CMAP to the fourth CMAP  ($\bar{x}$± s %) | | | The decrement from the first CMAP to the fifth CMAP  ($\bar{x}$± s %) | | |
| --- | --- | --- | --- | --- | --- | --- | --- |
|  |  | Amplitude decrement | Area decrement | *t / P* | Amplitude decrement | Area decrement | *t / P* |
| Trapezius | 421 | 2.345±2.516 | 3.594±3.578 | -2.978/0.003 | 2.369±2.486 | 3.863±3.908 | -5.524/<0.001 |
| ADM | 199 | 1.829±2.176 | 4.730±5.187 | -7.126/0.001 | 2.343±3.719 | 4.866±6.370 | -4.487/<0.001 |
| TAM | 54 | 3.932±3.344 | 5.122±4.027 | -3.472/<0.001 | 3.608±4.846 | 7.945±8.578 | -3.144/0.002 |

Note: ADM represents abductor digiti minimi, TAM represents tibialis anterior muscle.
